# A 17-Year-Old Female with Systemic Lupus Presents with Complex Movement Disorder: Possible Relationship with Antiribosomal P Antibodies

**DOI:** 10.1155/2013/590729

**Published:** 2013-05-08

**Authors:** Muhammed Emin Özcan, Meriç Adil Altınöz, Hasan Hüseyin Karadeli, Talip Asil, Abdulkadir Koçer

**Affiliations:** ^1^Department of Neurology, Bezmialem Vakıf University, Adnan Menderes Bulvarı, Vatan Caddesi, 34093 Fatih İstanbul, İstanbul, Turkey; ^2^Department of Molecular Biology, Haliç University, Büyükdere Caddesi No. 101, 34394 Mecidiyeköy İstanbul, İstanbul, Turkey; ^3^Department of Neurology, Medeniyet University, Dr. Erkin Caddesi, 34730 Kadıköy İstanbul, İstanbul, Turkey

## Abstract

Complex movement disorder is a relatively rare presentation of neurolupus. Antiphospholipid antibodies are associated with movement disorders likely via aberrant neuronal stimulation. Antiribosomal P antibodies have been previously associated with neuropsychiatric disorders but their correlation with movement disorder was not previously established. Our case report involves a 17-year-old Caucasian female patient positive for only antiribosomal P antibody and lupus anticoagulant who presented with a sudden onset of complex movement disorder. After complete cessation of physical signs with olanzapine, anticardiolipin and anti-**β**2 glycoprotein I antibodies became positive which indicates a likely discordance between movement disorder and antiphospholipid antibodies. This also indicates a potential causal role of antiribosomal P antibodies in inducing movement disorder.

## 1. Introduction

Systemic lupus erythematosus (SLE, lupus) is a complex, multisystem autoimmune disease involving connective tissue. The disease, like other autoimmune diseases, is more frequent in females in their mid-20s or early 30s (9 : 1) [1]. The rate of neurologic symptoms in SLE vary between 14% and 90% according to literature, with an average rate of 50% [2]. Approximately 10–20% of all cases with SLE are diagnosed during childhood. The female to male ratio is 9 : 1 in adulthood and 4 : 1 in puberty. The course of SLE in childhood is more aggressive and organ involvement, often brain and kidney, is more frequent [1]. Neurological involvement is much more frequent in pediatric lupus.

Neurological involvement in SLE is called “neurolupus” and in order of incidence it presents with stroke, headache, and epileptic seizures [3]. Movement disorders are a rare form of neurological involvement and often present with chorea [2, 4]. There are studies suggesting that cases with neurolupus especially in those who presented with movement disorders may be caused by antiphospholipid antibodies without any objective radiological findings in the brain [5, 6].

Our case is a patient with neurolupus that initially presented as a movement disorder. While the patient had complex movement disorder, she had no objective cranial MRI findings and positive antiribosomal P antibody and lupus anticoagulant tests. She tested negative for other antiphospholipid antibodies. The patient responded very well to Olanzapine but became positive for anti-*β*2 glycoprotein I and anticardiolipin IgG/IgM while in remission for neurological symptoms. This case is presented in order to inform clinicians involved in follow-up care for patients with neurolupus about the inconsistency between antiphospholipid antibodies and movement disorder. Additionally, the goal of this case report is to demonstrate that antiribosomal P antibodies binding to cellular membranes may cause physical symptoms.

## 2. Case Presentation

A 17-year-old female was admitted with a month-long history of involuntary movement bilaterally in hands and legs. The patient stated that the movements started one month ago and she also had occasionally involuntary movements of her mouth. The symptoms were mild in the beginning and gradually increased. The patient had dyskinesia around her mouth and choreic and occasionally choreathetoid movements in bilateral upper extremities. She had similar movements in the lower extremity. The patient could not stand still and had difficulty while walking. She had consulted a rheumatologist about pain, swelling, redness in the joints, redness on face, and sensitivity to sunlight one and half months ago. After lab tests and physical examination the patient was diagnosed with systemic lupus erythematosus and was on methylprednisolone 64 mg/day and hydroxychloroquine for 1 month. The patient was prediagnosed with central nervous system involvement of SLE and was hospitalized. The patient's cranial, cervical, and thoracic MRI results were normal. Detailed lab tests were ordered to rule out diagnoses other than SLE. The patient tested positive for anti-nuclear antibody (ANA) and anti-ds-DNA antibody tests. She was checked for all vasculitis indicators that may cause movement disorders by affecting the central nervous system. Because there are cases in literature that have complex movement disorders caused by the direct effect of anti-phospholipid antibodies, antiphospholipid antibody and antiribosomal P antibody tests were ordered. The patient tested positive for lupus anticoagulant and antiribosomal P antibody but negative for anticardiolipin IgG, IgM, and anti-*β*2 glycoprotein I IgG, IgM. The patient was administered Olanzapine 2.5 mg/day PO for her movement disorder. The patient responded very well to treatment and on the second day of treatment she did not have any symptoms of her movement disorder. The patient was discharged and antiphospholipid antibody and antiribosomal P antibody tests were repeated after 4 weeks and the patient tested negative for all tests except lupus anticoagulant and antiribosomal P antibody. The neurological examination of the patient 3 months after the hospitalization was normal. The patient had no signs of a movement disorder. Her Olanzapine was discontinued. The neurological examination was normal 2 weeks after the end of treatment. The patient was still positive for lupus anticoagulant and antiribosomal P antibody tests, but she also tested positive for anticardiolipin antibodies and anti-*β*2 glycoprotein I IgM which were previously negative.

## 3. Discussion

Patients with antiphospholipid antibody syndrome can have movement disorders. In a prior study antiphospholipid antibodies directly caused this effect by binding to neurons in basal ganglia [5]. The absence of radiological findings in the central nervous system strengthens the argument that direct cell stimulation can cause symptoms without the disruption of the blood-brain barrier. A study by Dale et al. showed that IgG antibodies bind directly to the neuronal cell membrane and this may be causing movement disorders like chorea that are related to antiphospholipid antibody syndrome and SLE. Our patient had movement disorder as an early symptom in SLE consistent with central nervous system involvement in SLE, called neurolupus. But cranial MRI studies of the patient showed no objective radiological findings. All lab tests that can be affected by SLE including anti phospholipid antibody and antiribosomal P antibody were evaluated. Anticardiolipin and anti-*β*2 glycoprotein I antibodies that are likely to cause this condition were negative at discharge and one month after discharge. Antiribosomal P antibody and lupus anticoagulant were positive each time. This indicates that antiribosomal P antibody and lupus anticoagulant are likley related to movement disorders that occur during the course of SLE.

Antiribosomal P antibodies were defined for the first time by Elkon et al. in 1985 [7]. They are antibodies directed against alanine rich phospho proteins called P0, P1, and P2 located in ribosomes inside the cell [7–9]. Antiribosomal P antibodies are specific to SLE [10]. The prevalence of positive antiribosomal P antibody among patients with SLE varies between 6% and 46%. It is less common in African-Americans and Caucasians and more common in Asians [11–13]. Although antiribosomal P antibodies are related to nephritis and hepatitis in the course of SLE [14, 15], a study by Bonfa et al. reexamined the association of antiribosomal P antibodies with neuropsychiatric symptoms of SLE [16]. Many studies for [17–20] and against [21–23] this study have been published since.

In our case, the absence of objective radiological and lab test findings to explain the complex movement disorder of the patient brought us to the conclusion that movement disorders in the course of SLE may be related to antiribosomal P antibodies. Lupus anticoagulants were also positive in our patient but in our literature review we did not come across any data regarding a relation between lupus anticoagulant and the neuronal membrane. Agius et al. published a case that initially presented with acute psychosis accompanied by chronic movements and of all the lab parameters only ribosomal P antibodies were positive [24]. Moreover in a study by Aldar et al. on 50 pediatric SLE patients, antiribosomal P antibodies were positive in 13 patients and only one of these patients had chorea [25]. However, there is no data regarding only antiribosomal P antibodies were positive and antiphospholipid antibodies were negative in this patient.

The patient's neurological examination was normal after 3 months but antiribosomal P antibody and lupus anticoagulant were positive. Additionally, anticardiolipin antibody and anti-*β*2 glycoprotein I IgM that were previously negative were positive. These results suggest that movement disorders in neurolupus can occur independent of anti phospholipid antibodies.

Although the relationship between anti-phospholipid antibodies and movement disorders has been shown in prior studies, the fact that antiribosomal P antibodies bind directly to the neuron membrane [26] suggests that anti-phospholipid antibody positivity may be an epiphenomenon.

The remission of all physical symptoms in our patient with Olanzapine treatment may suggest that the disease, at least in the early stages, begins only with cell signalization due to membrane-antibody interaction without major neuron damage. Olanzapine shows its affects via 5HT2 and D2 receptors [27]. Since hypothetical antibodies in neurolupus cause movement disorders by binding to dopaminergic neurons, Olanzapine may have been selectively beneficial for our patient. According to studies, Olanzapine suppresses microinflammation and contributes to preventing the inflammation on a genetic level with chronic use [28, 29]. These effects may have contributed to the clinical benefit of Olanzapine. However, further studies on this subject including more patients are needed. The clinical importance of antiribosomal P antibodies is still under discussion. Although its relationship with neuropsychiatric findings in SLE is shown clearly through various studies [16, 20], whether the positive antiribosomal P antibody in SLE has a predictive value for long-term neuropsychiatric symptoms is yet to be answered. The relationship of antiribosomal P antibodies with movement disorders that occur in the course of SLE is not clearly known. Since this relationship is not certain, antiribosomal P antibody tests are not always routinely ordered. Routinely checking SLE patients with movement disorders for antiribosomal P antibody positivity may be beneficial in understanding this relationship. Large scaled studies are needed to enlighten this subject. 
